# Supplementary material for: Two coacting shadow enhancers regulate twin of eyeless expression during early Drosophila development
Source: Genetics. 2024 Nov 28;229(1):iyae176. doi: 10.1093/genetics/iyae176 (PMC11708921; doi:10.1093/genetics/iyae176)
Supplement: iyae176_Supplementary_Data [file iyae176_supplementary_data.zip › RD Supplemental_Material_Legends_GENETICS-2024-307563.docx]

**Supplementary Information**

**Figure S1. Quantitative analysis of embryos**

Simple intensity plot across the dorsal-ventral axis of a representative embryo. **(A)** Depiction of binary mask of the embryo of interest. The red line indicates the full egg length, from the anterior-most position (0% AP) to the posterior-most position (100% AP). The blue line indicates the line of interest, which was taken at 25% AP and runs the length of the embryo at the designated 25% AP position from the dorsal-most point (0% DV) to the ventral-most point (100% DV). **(B)** Plot of intensity from 0% to 100% of the dorsal-ventral axis, at 25% of the AP axis. Inset shows the line across the embryo being plotted. Inset shows the line across the embryo being plotted.

**Figure S2. Patser TFBS predictions**

Spatial arrangement of Patser predicted transcription factor binding sites within each putative *Drosophila melanogaster* enhancer. Bars pointing up or down represent binding sites on the forward or reverse DNA strand, respectively. Bar width corresponds to the length of the binding site Position Weight Matrix (PWM) sequence used for binding site predictions. Bar height is proportional to the score for each site, with a longer bar representing a higher similarity score for the predicted binding site to the PWM.

**Table S1. Patser TFBS summary**

Transcription factor binding sites predicted by Patser for each putative enhancer zone. BCD – bicoid, CAD – caudal, D - Dichaete, Dl – dorsal, HB – Hunchback, KNI – knirps, KR – Kruppel, TWI – twist.

**Table S2. Parameter values for models with minimum error**

Parameter values that resulted in the best model fit, as measured by AIC, for each enhancer zone. Parameters in sheet 1 correspond to the optimized parameter values for the best performing model on the zone 1 experimental data, as shown in Fig. 6g. Parameters in sheet 2 correspond to the optimized parameter values for the best performing model on the zone 2 experimental data, as shown in Fig. 6g.

**Figure S3. Epigenetic profile of *toy* genomic region**

The 48.5kb genomic region (chr4: 962259-1010757) around the *twin-of-eyeless*(*toy*) gene visualized using the WashU Epigenome Browser (<http://epigenomegateway.wustl.edu/browser/>) in the top panel and the HiGlass genome browser (<https://higlass.io>) (Kerpedjiev et al. 2018) in the bottom panel. The four putative enhancer zones selected for *­in vivo* analysis in our study are colored coded (1 – light blue, 2 – purple, 4 – red, 7 – orange) and numbered between the top and bottom panels. Epigenetic tracks were sourced from different studies focusing on specific embryonic stages. Enhancer predictions for stages 2-4 and 5-13 were downloaded from Enhancer Atlas 2.0 (Gao and Qian 2020). H3K27 acetylation data (Zenk et al. 2021; Brennan et al. 2023) shows a number of broad peaks surrounding the enhancers, but not directly within them. ATAC-seq data (Brennan et al. 2023) shows extensive areas of open chromatin, with several sharp peaks, particularly in zones 1 and 7, close to the transcriptional start sites for *fuss* and *toy* respectively. Clear peaks of CTCF (Roy et al. 2010; Nègre et al. 2011; Consortium 2012) and Zelda (Brennan et al. 2023), both architectural proteins known to involved in chromatin looping, are also present in zones 1 and 7. This chromatin looping is supported by Micro-C visualization (bottom panel), with the edges of a region of high contact probability (red rectangle) between zone 1 and 7, with the anchor sites correlating with the CTCF/Zelda peaks described above.

**Figure S4. Comparative analysis of model performance**

Spatial arrangement of the 10 Transcription Factor binding sites (TFBSs) predicted by a PWM-based algorithm in zone 1 (**A**) and zone 2 (**B**) that were used to construct thermodynamic-based models for each of the two enhancers. For each enhancer, the two strongest BCD (pink), CAD (blue), HB (purple), KNI (brown) and KR (red) binding sites are selected. The five TFBSs included in the best performing models, as measured by Akaike Information Criterion (AIC), for zone 1 (**C**) and 2 (**D**) are shown. The minimum root mean square error (RMSE) found for each of the best models when fit to the experimental expression data for zone 1 (**E**) and 2 (**F**) is shown with a red line. For each zone, the five TFBSs in the best performing models were randomly rearranged 100 times, model parameters were refit and the RMSE calculated (black dots). Not a single rearrangement in either zone resulted in an RMSE below that obtained from the original TFBS arrangement. (**G**) Model predictions for zone 1 (black line), zone 2 (blue line), concatenated zone 1 and zone (red line), and *toy* expression data (black dots) are shown. The x-axis represents the position along the Anterior-Posterior (AP) axis from 10 – 90% and the y-axis represents normalized expression level.

**Figure S5. Models of BICOID-driven enhancers**

Spatial arrangement of the previously characterized Transcription Factor binding sites (TFBSs) in the *even-skipped* stripe 2 (eve stripe 2) enhancer (**A**) and the giant 23 (gt 23) enhancer (**B**); BCD (pink), HB (purple), GT (blue) and KR (red). For the eve stripe 2 (**C**) and gt 23 (**D**) enhancers, the thermodynamic-based model parameters were fit as described in Methods (red line), model predictions plotted against expression data (black dots) obtained from BDTNP at timepoint 2 (70 mins), and the minimum root mean square error (RMSE) calculated. Resulting RMSEs were 0.02 for eve stripe 2 and 0.09 for gt 23.

**Table S3. Predicted ZELDA binding sites**

All bioinformatically predicted ZELDA (ZLD) binding sites in zones 1 (light blue), 2 (purple), 4 (red), and 7 (orange) that resulted in a positive match score are shown.  Note that the top five sites are found in zones 1 and 2 , while only four of the 25 total sites are found in zones 4 or 7.

**SI References**

Brennan, K. J., M. Weilert, S. Krueger, A. Pampari, H. Y. Liu, A. W. H. Yang, J. A. Morrison, T. R. Hughes, C. A. Rushlow, A. Kundaje, and J. Zeitlinger. 2023. "Chromatin accessibility in the Drosophila embryo is determined by transcription factor pioneering and enhancer activation." *Dev Cell* 58 (19): 1898-1916.e9. <https://doi.org/10.1016/j.devcel.2023.07.007>.

Consortium, ENCODE Project. 2012. "An integrated encyclopedia of DNA elements in the human genome." *Nature* 489 (7414): 57-74. <https://doi.org/10.1038/nature11247>.

Gao, T., and J. Qian. 2020. "EnhancerAtlas 2.0: an updated resource with enhancer annotation in 586 tissue/cell types across nine species." *Nucleic Acids Res* 48 (D1): D58-D64. <https://doi.org/10.1093/nar/gkz980>.

Nègre, N., C. D. Brown, L. Ma, C. A. Bristow, S. W. Miller, U. Wagner, P. Kheradpour, M. L. Eaton, P. Loriaux, R. Sealfon, Z. Li, H. Ishii, R. F. Spokony, J. Chen, L. Hwang, C. Cheng, R. P. Auburn, M. B. Davis, M. Domanus, P. K. Shah, C. A. Morrison, J. Zieba, S. Suchy, L. Senderowicz, A. Victorsen, N. A. Bild, A. J. Grundstad, D. Hanley, D. M. MacAlpine, M. Mannervik, K. Venken, H. Bellen, R. White, M. Gerstein, S. Russell, R. L. Grossman, B. Ren, J. W. Posakony, M. Kellis, and K. P. White. 2011. "A cis-regulatory map of the Drosophila genome." *Nature* 471 (7339): 527-31. <https://doi.org/10.1038/nature09990>.

Roy, S., J. Ernst, P. V. Kharchenko, P. Kheradpour, N. Negre, M. L. Eaton, J. M. Landolin, C. A. Bristow, L. Ma, M. F. Lin, S. Washietl, B. I. Arshinoff, F. Ay, P. E. Meyer, N. Robine, N. L. Washington, L. Di Stefano, E. Berezikov, C. D. Brown, R. Candeias, J. W. Carlson, A. Carr, I. Jungreis, D. Marbach, R. Sealfon, M. Y. Tolstorukov, S. Will, A. A. Alekseyenko, C. Artieri, B. W. Booth, A. N. Brooks, Q. Dai, C. A. Davis, M. O. Duff, X. Feng, A. A. Gorchakov, T. Gu, J. G. Henikoff, P. Kapranov, R. Li, H. K. MacAlpine, J. Malone, A. Minoda, J. Nordman, K. Okamura, M. Perry, S. K. Powell, N. C. Riddle, A. Sakai, A. Samsonova, J. E. Sandler, Y. B. Schwartz, N. Sher, R. Spokony, D. Sturgill, M. van Baren, K. H. Wan, L. Yang, C. Yu, E. Feingold, P. Good, M. Guyer, R. Lowdon, K. Ahmad, J. Andrews, B. Berger, S. E. Brenner, M. R. Brent, L. Cherbas, S. C. Elgin, T. R. Gingeras, R. Grossman, R. A. Hoskins, T. C. Kaufman, W. Kent, M. I. Kuroda, T. Orr-Weaver, N. Perrimon, V. Pirrotta, J. W. Posakony, B. Ren, S. Russell, P. Cherbas, B. R. Graveley, S. Lewis, G. Micklem, B. Oliver, P. J. Park, S. E. Celniker, S. Henikoff, G. H. Karpen, E. C. Lai, D. M. MacAlpine, L. D. Stein, K. P. White, and M. Kellis. 2010. "Identification of functional elements and regulatory circuits by Drosophila modENCODE." *Science* 330 (6012): 1787-97. <https://doi.org/10.1126/science.1198374>.

Zenk, F., Y. Zhan, P. Kos, E. Löser, N. Atinbayeva, M. Schächtle, G. Tiana, L. Giorgetti, and N. Iovino. 2021. "HP1 drives de novo 3D genome reorganization in early Drosophila embryos." *Nature* 593 (7858): 289-293. <https://doi.org/10.1038/s41586-021-03460-z>.
