# Supplementary material for: Two coacting shadow enhancers regulate twin of eyeless expression during early Drosophila development
Source: Genetics. 2024 Nov 28;229(1):iyae176. doi: 10.1093/genetics/iyae176 (PMC11708921; doi:10.1093/genetics/iyae176)
Supplement: iyae176_Supplementary_Data [file iyae176_supplementary_data.zip › Table_S1_GENETICS-2024-307563.pdf]

Zone	BCD	CAD	D	DL	HB	KNI	KR	TWI	Total	Sequence length (bp)	Density (bp/TFBS)
**Zone 1**	8	11	13	11	13	15	18	10	99	2304	23.27
**Zone 2**	6	11	9	8	3	4	11	6	58	1243	21.43
**Zone 3**	1	13	11	3	0	5	6	10	49	1341	27.36
**Zone 4**	4	6	7	7	4	0	6	7	41	1087	26.51
**Zone 5**	2	4	4	3	3	0	5	11	32	551	17.21
**Zone 6**	3	1	6	1	2	2	1	3	19	688	36.21
**Zone 7**	4	2	2	0	3	1	5	6	23	806	35.04
**Zone 8**	9	11	5	8	7	9	6	5	60	1094	18.23
**Zone 9**	1	1	2	0	0	0	2	6	12	309	25.75
**Zone 10**	2	2	1	3	0	1	6	2	17	529	31.12
**Zone 11**	3	11	6	1	1	4	3	8	37	634	17.14
**Zone 12**	5	5	2	5	4	4	5	2	32	817	25.53
**Zone 13**	3	6	5	2	3	5	8	2	34	764	22.47
**Zone 14**	3	2	5	5	0	4	1	9	29	883	30.45
**Zone 15**	2	4	3	0	1	0	3	0	13	280	21.54
**Zone 16**	6	19	12	7	14	8	13	9	88	1861	21.15
