# Supplementary material for: Two coacting shadow enhancers regulate twin of eyeless expression during early Drosophila development
Source: Genetics. 2024 Nov 28;229(1):iyae176. doi: 10.1093/genetics/iyae176 (PMC11708921; doi:10.1093/genetics/iyae176)
Supplement: iyae176_Supplementary_Data [file iyae176_supplementary_data.zip › Table_S3_GENETICS-2024-307563.pdf]

Sequence	Zone	Position	Match Score	P value
AAGCGCAGTTAGGTC	2	934	5.6418	0.0003
TTGTACAGGTATGTA	1	373	5.6335	0.0002
AGTAACAGGTATCTG	1	1321	5.3502	0.0002
TTACTCAGTTAGTGG	2	1146	5.3360	0.0002
GGTATCTGGTAGCCC	1	1328	4.7134	0.0002
TGTTTCTGGTAGAAG	4	247	4.3866	0.0001
AGGGTCAGGTCCAGG	4	296	3.9563	0.0002
TGACGTAGGTATCGG	7	706	3.8512	0.0002
TTTATCAGTTACCCG	2	1131	3.7308	0.0003
GTGCGCAGTTATTTA	2	852	3.6205	0.0005
CTGCGCAGTTATTAA	4	663	2.9158	0.0003
CTCGGCTGGTAATCC	1	858	2.9131	0.0006
GAGCTCATGTATTAC	1	504	2.5632	0.0007
GATCGCAGGAATTTT	1	882	2.3529	0.0011
GTTTTAAGGTAAACT	2	1113	2.0759	0.0009
GTATTCGGGTAACGG	1	1830	1.8541	0.0011
GCACACATGTACATA	2	557	1.7151	0.0011
GAATTTAGGTAACGA	1	439	1.1845	0.0020
ACAGGTATGTAGCGA	1	337	0.9724	0.0021
TTTTTCAGATATGTG	1	1236	0.9246	0.0016
ACTATCAGATACCCG	1	2301	0.6876	0.0016
ATTTTCTGGTATGTC	1	1286	0.4941	0.0018
ACAATCTGGTATCGG	1	172	0.1701	0.0019
GGTTGAAGGAAGAAT	1	793	0.0542	0.0026
TGTAACAGGTTGAAG	1	786	0.0247	0.0026
