# Supplementary figures and images for: Two coacting shadow enhancers regulate twin of eyeless expression during early Drosophila development
Source: Genetics. 2024 Nov 28;229(1):iyae176. doi: 10.1093/genetics/iyae176 (PMC11708921; doi:10.1093/genetics/iyae176)

**A**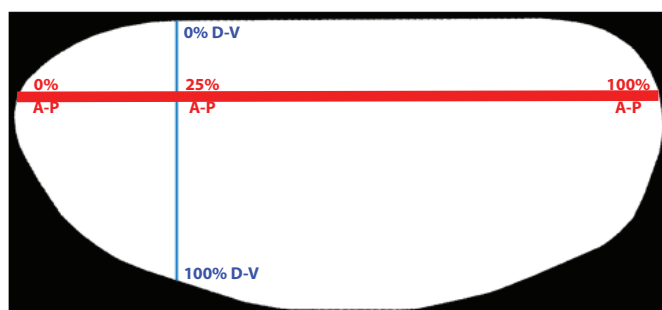**B**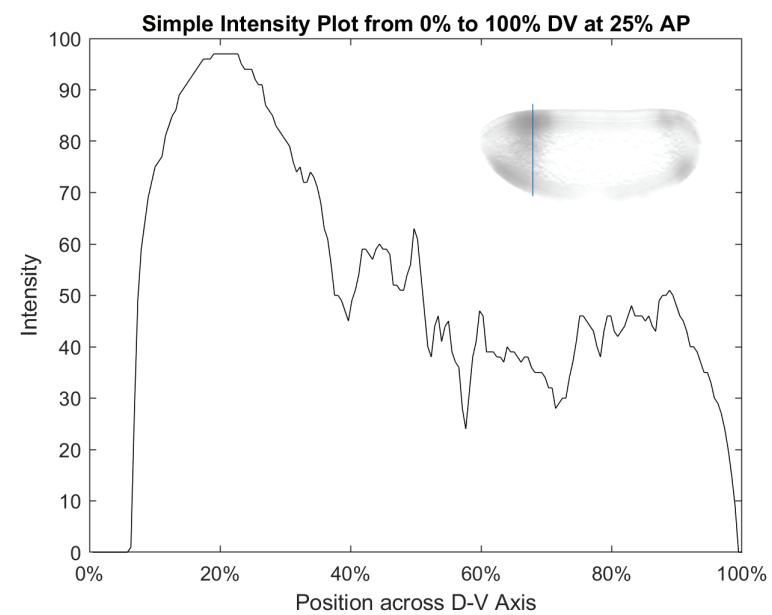

Supplement: iyae176_Supplementary_Data [file iyae176_supplementary_data.zip › Figure_S1_GENETICS-2024-307563.pdf]

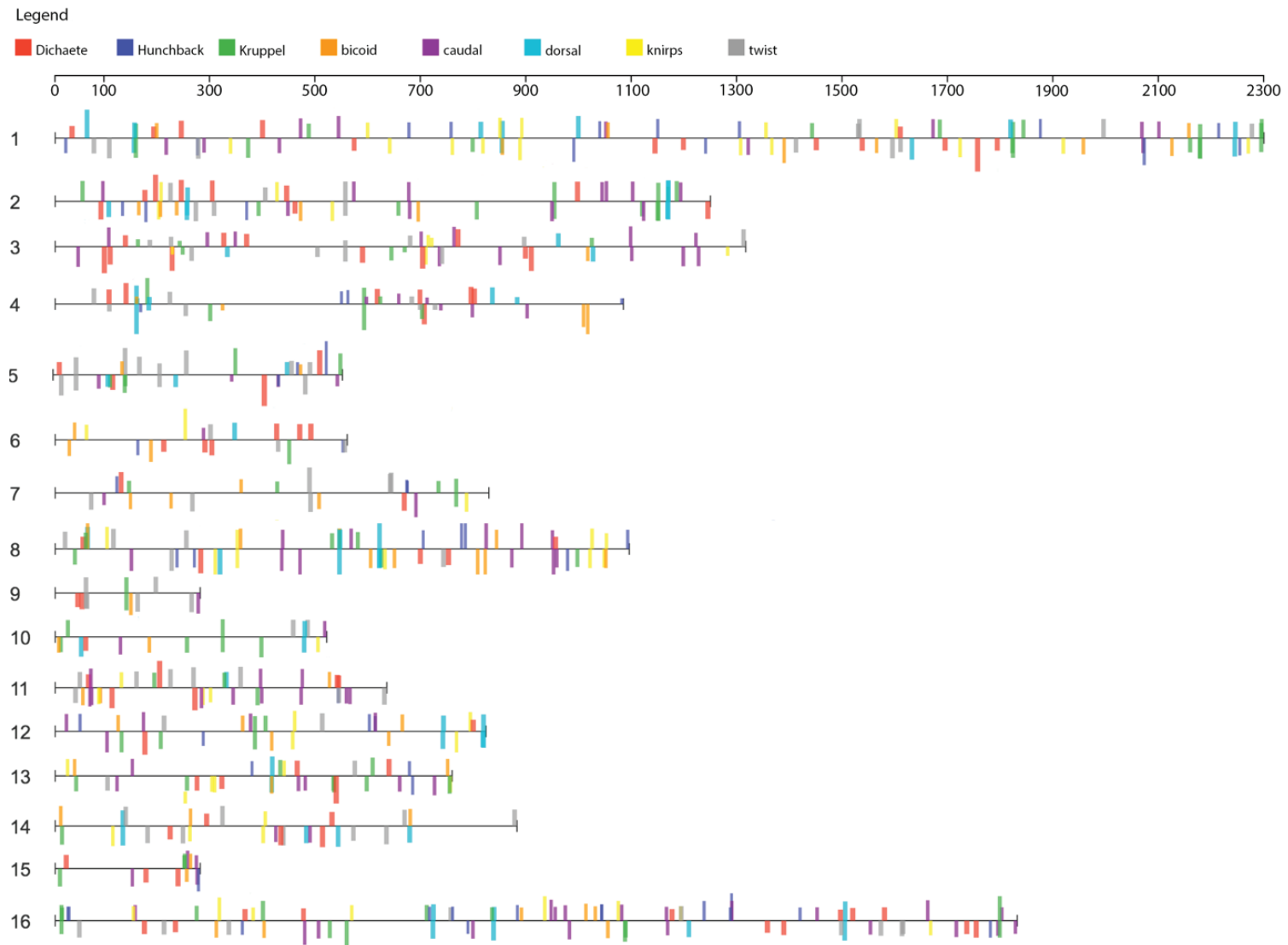

Supplement: iyae176_Supplementary_Data [file iyae176_supplementary_data.zip › Figure_S2_GENETICS-2024-307563.pdf]

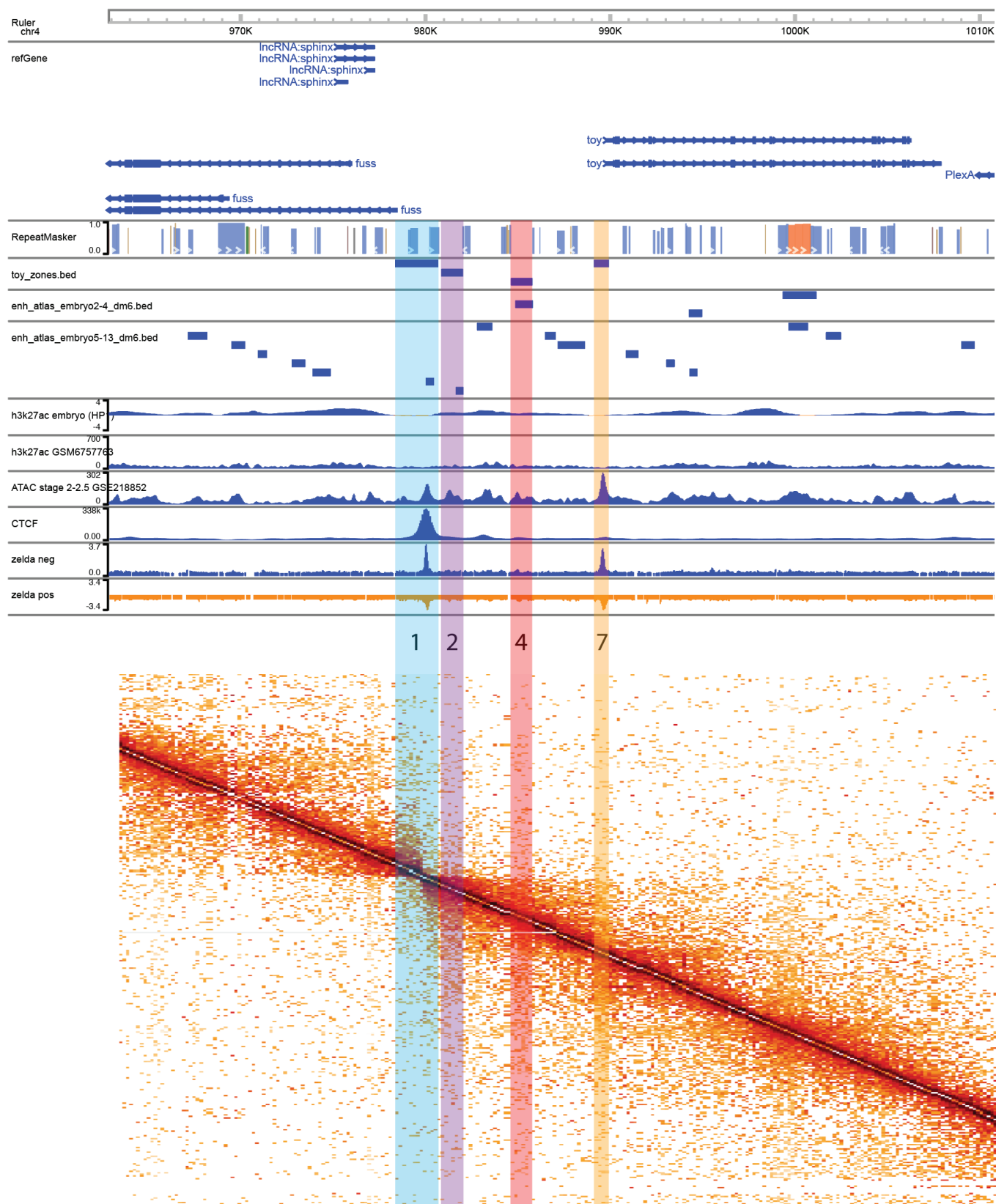

Supplement: iyae176_Supplementary_Data [file iyae176_supplementary_data.zip › Figure_S3_GENETICS-2024-307563.pdf]

Zone 1

Zone 2

**A**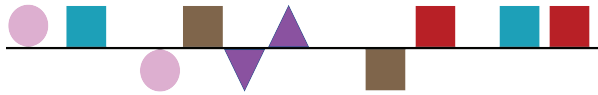**B**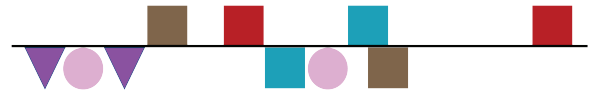**C**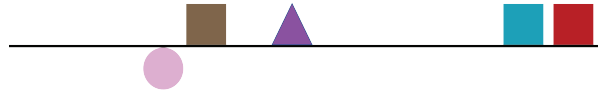**D**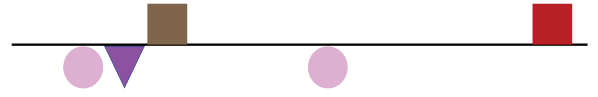**E**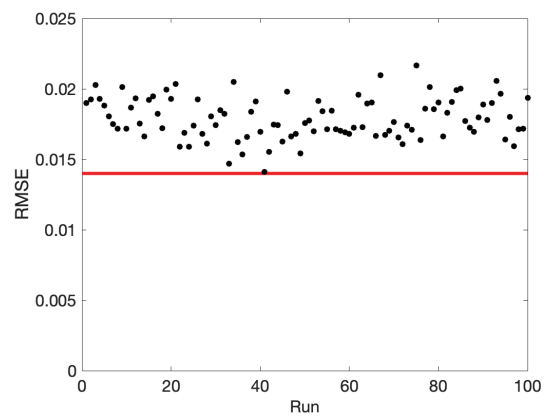**F**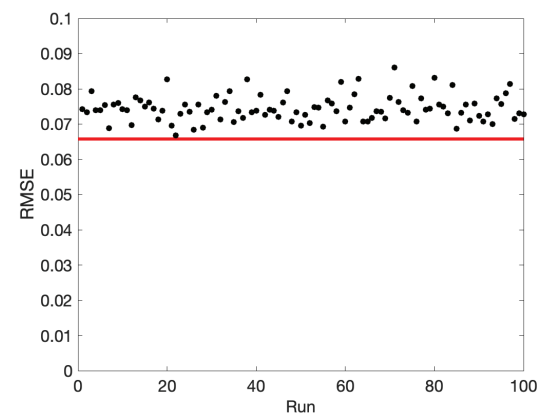**G**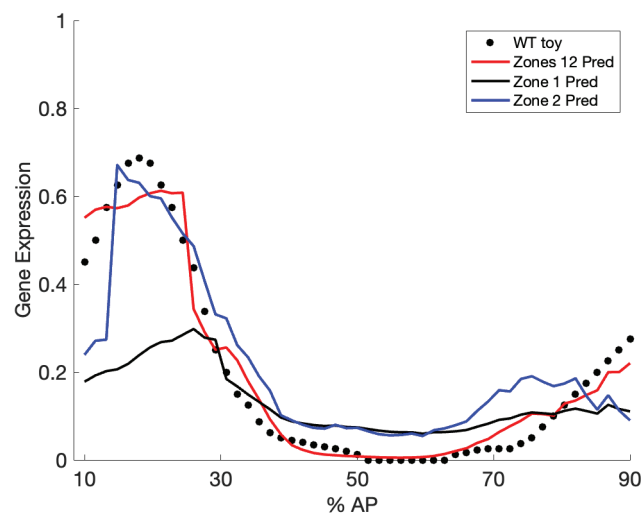

Supplement: iyae176_Supplementary_Data [file iyae176_supplementary_data.zip › Figure_S4_GENETICS-2024-307563.pdf]

eve stripe 2

**A**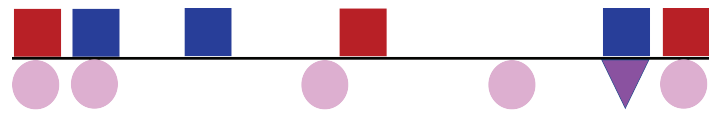

gt 23

**B**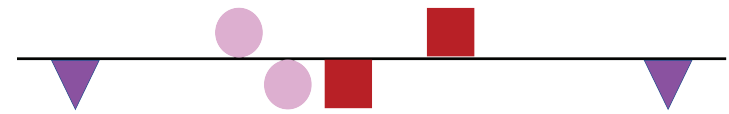**C**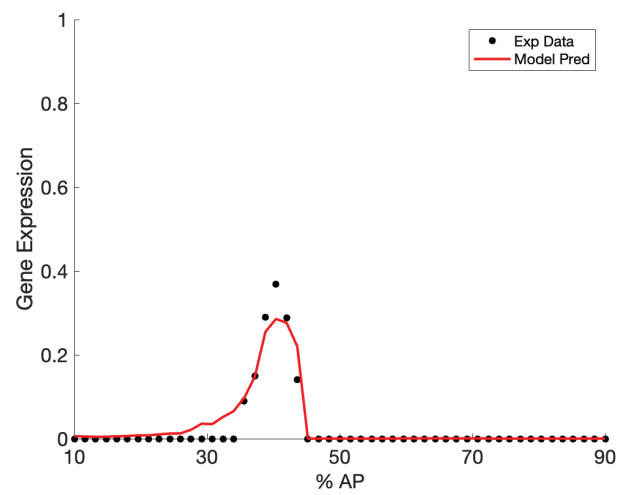**D**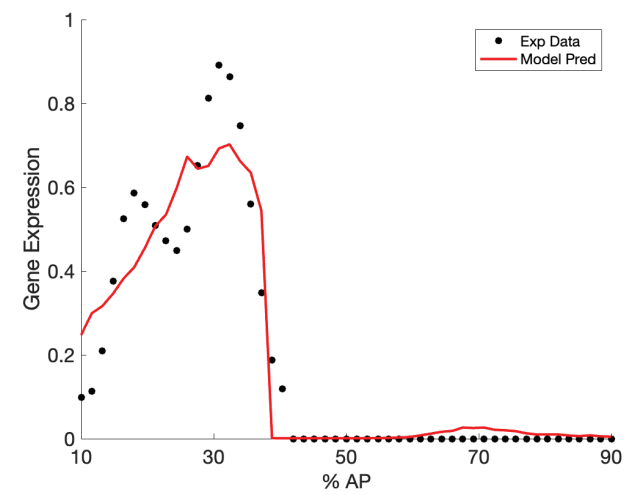

Supplement: iyae176_Supplementary_Data [file iyae176_supplementary_data.zip › Figure_S5_GENETICS-2024-307563.pdf]
